# Expression of uncoupling proteins-1, -2 and -3 mRNA is induced by an adenocarcinoma-derived lipid-mobilizing factor

**DOI:** 10.1038/sj.bjc.6600101

**Published:** 2002-02-12

**Authors:** C Bing, S T Russell, E E Beckett, P Collins, S Taylor, R Barraclough, M J Tisdale, G Williams

**Affiliations:** Diabetes and Endocrinology Research Group, Department of Medicine, University of Liverpool, Liverpool L69 3G, UK; Pharmaceutical Sciences Research Institute, Aston University, Birmingham B4 7ET, UK; School of Biological Science, University of Liverpool, Liverpool L69 72B, UK

**Keywords:** lipid-mobilizing factor, uncoupling proteins, cancer cachexia

## Abstract

The abnormalities of lipid metabolism observed in cancer cachexia may be induced by a lipid-mobilizing factor produced by adenocarcinomas. The specific molecules and metabolic pathways that mediate the actions of lipid-mobilizing factor are not known. The mitochondrial uncoupling proteins-1, -2 and -3 are suggested to play essential roles in energy dissipation and disposal of excess lipid. Here, we studied the effects of lipid-mobilizing factor on the expression of uncoupling proteins-1, -2 and -3 in normal mice. Lipid-mobilizing factor isolated from the urine of cancer patients was injected intravenously into mice over a 52-h period, while vehicle was similarly given to controls. Lipid-mobilizing factor caused significant reductions in body weight (−10%, *P*=0.03) and fat mass (−20%, *P*<0.01) accompanied by a marked decrease in plasma leptin (−59%, *P*<0.01) and heavy lipid deposition in the liver. In brown adipose tissue, uncoupling protein-1 mRNA levels were elevated in lipid-mobilizing factor-treated mice (+96%, *P*<0.01), as were uncoupling proteins-2 and -3 (+57% and +37%, both *P*<0.05). Lipid-mobilizing factor increased uncoupling protein-2 mRNA in both skeletal muscle (+146%, *P*<0.05) and liver (+142%, *P*=0.03). The protein levels of uncoupling protein-1 in brown adipose tissue and uncoupling protein-2 in liver were also increased with lipid-mobilizing factor administration (+49% and +67%, both *P*=0.02). Upregulation by lipid-mobilizing factor of uncoupling proteins-1, -2 and -3 in brown adipose tissue, and of uncoupling protein-2 in skeletal muscle and liver, suggests that these uncoupling proteins may serve to utilize excess lipid mobilized during fat catabolism in cancer cachexia.

*British Journal of Cancer* (2002) **86**, 612–618. DOI: 10.1038/sj/bjc/6600101 www.bjcancer.com

© 2002 Cancer Research UK

## 

Body fat depletion due to enhanced lipid mobilization is a major component of weight loss in cachectic cancer patients (Shaw and Wolfe, 1987; Puccio and Nathanson, 1997). Cachexia can occur with a small tumour burden, suggesting that tumour-derived factors catabolize body fat, but their nature and mode of action have remained elusive.

One putative mediator is a lipid-mobilizing factor (LMF), a 43-kD protein with homology to Zn-α_2_-glycoprotein, which was first isolated from the murine MAC16 adenocarcinoma and subsequently from the urine of cachectic patients with gastrointestinal and pancreatic cancers (Todorov *et al*, 1998). Administration of human LMF to normal mice over 3 days causes a massive (40%) reductions in body weight and fat, without changes in muscle mass or in food or water intake (Hirai *et al*, 1998). LMF enhances lipolysis in adipocytes and increases serum free fatty acid (FFA) levels, by activating hormone-sensitive lipase (HSL) through increased intracellular cyclic AMP levels (Hirai *et al*, 1998). Notably, LMF is also implicated in human cancer cachexia, as it is found only in patients with gastrointestinal tumours that are complicated by weight loss (Groundwater *et al*, 1999).

The fate of the FFA and glycerol liberated from lipolysis induced by LMF is not known. These products must be catabolized and/or resynthesized in other sites to form triglyceride. Evidence is emerging that the uncoupling proteins (UCP-1, -2 and -3), members of the mitochondrial carrier family, may provide mechanisms for disposing of excess FFA (Samec *et al*, 1998a; Ricquier and Bouillaud, 2000). UCP-1 is expressed exclusively in brown adipose tissue (BAT), a major heat-producing tissue in rodents and human neonates, where it dissipates the proton electrochemical gradient across the inner mitochondrial membrane; this uncoupling increases heat production instead of generating ATP from the oxidation of FFA (Ricquier and Bouillaud, 2000). UCP-1 expression is stimulated by the sympathetic nervous system via β_3_ adrenoceptors, being induced by cold exposure and β_3_ adrenoceptor agonists, but falls on fasting (Champigny and Ricquier, 1990; Bing *et al*, 1997, 1998). UCP-2 and -3 are two newly-described homologues of UCP-1. UCP-2 is widely expressed in most tissues including adipose tissue, muscle, heart and liver (Fleury *et al*, 1997), while UCP-3 mRNA is preferentially expressed in skeletal muscle and BAT (Boss *et al*, 1997b). Overexpression of UCP-2 and -3 reduces the mitochondrial membrane potential in yeast, consistent with uncoupling activity (Fleury *et al*, 1997; Gimeno *et al*, 1997); they are therefore candidate thermogenic mediators in tissues that lack UCP-1. There is some indirect evidence that they are involved in heat production *in vivo*: adipose tissue UCP-2 mRNA levels correlate with resting metabolic rate in obese women (Barbe *et al*, 1998), while transgenic overexpression of UCP-3 in skeletal muscle of mice stimulates thermogenesis and markedly reduces body weight and fat (Clapham *et al*, 2000).

Paradoxically, however, muscle UCP-2 and -3 expression is increased during fasting when energy expenditure falls (Weigle *et al*, 1998), raising the possibility that UCP-2 and -3 serve other physiological roles. Suggested alternative functions include the regulation of lipid (especially FFA) metabolism, as various situations that raise circulating levels of FFA increase both UCP-2 and -3 expression in white fat and skeletal muscle (Boss *et al*, 2000). These conditions include fasting, high-fat feeding and intralipid infusion in rodents (Boss *et al*, 1997a; Fleury *et al*, 1997; Matsuda *et al*, 1997; Weigle *et al*, 1998) and obesity, type-2 diabetes and intralipid infusion in man (Millet *et al*, 1997; Bao *et al*, 1999; Khalfallah *et al*, 2000; Nisoli *et al*, 2000). Moreover, *in vitro,* FFA up-regulate UCP-2 in pre-adipocytes and hepatocytes, and UCP-3 in muscle cells (Samec *et al*, 1998b; Cortez-Pinto *et al*, 1999; Reilly and Thompson, 2000). Conversely, inhibition of mitochondrial β-oxidation of fatty acids prevents upregulation of UCP-2 and -3 during fasting in the soleus muscle in rats (Hwang and Lane, 1999).

These observations suggest that UCP-2 and -3 help to utilize and dispose of excess lipid. They are therefore strong candidates for removing lipolytic products generated by LMF in cancer cachexia. This hypothesis is supported by our recent findings of increased expression of UCP-1 in BAT, and of UCP-2 and -3 in skeletal muscle in mice bearing the MAC16-tumour, which produces LMF (Bing *et al*, 2000). However, it is not known whether UCP expression is induced by LMF or by other MAC16 tumour products, which include a proteolysis-inducing factor (PIF). This study aimed to verify whether administration of LMF stimulates expression of UCP-1, -2 and -3 in normal mice.

## MATERIALS AND METHODS

### Animals

Male NMRI mice (30–35 g) from the inbred colony at Aston University were housed at an ambient temperature of 22±2°C under a 12 : 12 h light–dark cycle (lights on at 0700 h) and fed on standard chow (SDS economy breeder; Lillico, Wonham Mill, Bletchworth, Surrey, UK), with tap water *ad libitum*. Food intake and body weight were monitored daily. All studies were conducted according to the UKCCCR Guidelines for the care and use of laboratory animals.

### LMF purification

**Figure 1:**
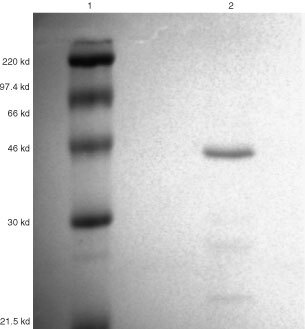
Twelve per cent SDS polyacrylamide gel electrophoresis of human LMF. Lane 1, molecular weight markers; Lane 2, human LMF purified from the urine of cachectic cancer patients, showing expected molecular weight of 43 kDa. Detection was by Coomassie Blue staining.

LMF was purified from the urine of cachectic cancer patients who had suffered >10% weight loss, and was kindly provided by Professor K Fearon, Department of Surgery, Edinburgh Royal Infirmary, Scotland. Urine was diluted with four parts of 10 mM Tris HCl (pH 8.0) before addition of DEAE cellulose (Whatman International Ltd, Kent, UK) 2 g per litre of diluted urine, previously equilibrated in 100 mM Tris HCl (pH 8.0) for 5 min and then in 10 mM Tris HCl (pH 8.0) for 5 min, with stirring for 2 h at 4°C. The DEAE cellulose was recovered by low-speed centrifugation (4500 r.p.m. for 3 min) and LMF was eluted by resuspension (twice) in 0.3 M NaCl in 10 mM Tris HCl (pH 8.0). The eluate was equilibrated and concentrated to 1 ml by ultrafiltration against phosphate-buffered saline (PBS) in an Amicon filtration cell containing a membrane filter with a molecular weight cut-off of 10 kDa. Further purification was achieved using a Resource-Iso HPLC column (Pharmacia Biotech, St Albans, Herts., UK), employing a decreasing (NH_4_)_2_SO_4_ concentration from 1.5 M. Active fractions containing LMF eluted at 0.6 M (NH_4_)_2_SO_4_ and were desalted before use by washing five times against PBS using an Amicon filtration cell. Biological activity was determined by glycerol release from mouse epididymal adipocytes, as previously described (Hirai *et al*, 1998). The LMF was purified to a single band of M_r_ 43 kDa ( Figure 1

).

### LMF treatment

Repeated intravenous injections of LMF (8 μg) were given to one group of mice (*n*=6), while the weight-matched controls (*n*=6) were similarly injected with PBS at the same time-points: 0, 7, 11, 24, 32 and 48 h. Food intake and body weight were monitored before each injection, and core temperature was recorded at 52 h before the mice were killed by cervical dislocaton. Blood was removed by cardiac puncture and plasma was separated and stored at −40°C until assay. The interscapular BAT, gonadal fat pads, gastrocnemius muscle and liver were dissected, snap-frozen in liquid nitrogen and stored at −80°C until extraction of RNA.

### Liver histology

Liver was sectioned using a cryostat. Five-μm frozen sections were stained with haematoxylin and eosin, and compared with other sections stained with Oil Red O to demonstrate triglyceride deposition. Sections were rinsed in water, and then 60% isopropyl alcohol, and stained with 1% Oil Red O for 10 min; sections were then rinsed briefly again as above, and counterstained with Harris haematoxylin for 1 min. Finally, sections were washed in water, then mounted in Aquamount (BDH, Leicester, UK).

### Immunohistochemistry for caspase 3

This was performed to determine the extent of apoptosis in liver. Five-μm cryostat sections were first fixed on glass slides with 4% (w v^−1^) paraformaldehyde. Slides were rinsed three times in PBS, exposed to 5 μg ml^−1^ of rabbit anti-caspase 3 (R&D Systems, Abingdon, Oxfordshire, UK) and incubated at room temperature for 1 h. After three further rinses in PBS, biotin-labelled anti-rabbit IgG was applied to the slides for 30 min. Slides were rinsed in PBS and incubated with horseradish peroxidase-conjugated avidin (Amersham Pharmacia Biotech; Little Chalfont, Buckinghamshire, UK) for 30 min, and stained with 3,3′-diaminobenzidine for 5 min. Sections were counterstained with Mayer's haematoxylin for 2 min, dehydrated and mounted with Aquamount.

### Assays

Plasma leptin concentrations were measured using a mouse leptin ELISA kit (Crystal Chemicals, Chicago, USA). Plasma glucose was measured using a glucose oxidase-based kit (Sigma; Poole, Dorset, UK), and plasma glycerol and free fatty acids were determined using enzymatic colorimetric assay kits, respectively from Sigma and Wako Chemicals (Neuss, Germany). Skeletal muscle and liver triglyceride concentrations were measured with a kit (Sigma). The tissue (50 mg) was homogenized in 500 μl PBS with 0.1% BSA and then centrifuged for 5 min at 2000 *g*; triglyceride assay was performed on the supernatant.

### Northern blotting

Total RNA was extracted from BAT and gastrocnemius muscle using Tri-reagent (Sigma) and the RNA concentration determined from the absorbance at 260 nm. Aliquots of 20 μg were size-fractionated on a 1% agarose-formaldehyde gel, blotted on to a positively-charged membrane (Boehringer Mannheim, Lewes, Sussex, UK) and then cross-linked under UV light. UCP-1, -2 and -3 mRNA were detected by Northern blotting in conjunction with the chemiluminescence method. The membranes were pre-hybridized in Easyhyb solution (Boehringer Mannheim) at 42°C for 1 h and hybridized in the same solution with a digoxigenin-labelled 32-mer antisense oligonucleotide probe for mouse UCP-1 (Trayhurn and Duncan, 1994), or digoxigenin-labelled 30-mer oligonucleotide probes for mouse UCP-2 and -3 (Bing *et al*, 2000). Each blot was stripped and re-probed for 18S rRNA with a 31-mer digoxigenin-labelled oligonucleotide, as previously described (Trayhurn *et al*, 1995). Autoradiographs were quantitated by densitometry with image-analysis (AIS System, Imaging Technology, Brock University, St Catharine's, Ontario, Canada). The abundance of mRNA was expressed as the ratio of UCP mRNA/18S rRNA signals.

### Quantitative RT*–*PCR

Since the signal was too low to be detected by Nothern blotting, liver UCP-2 mRNA was quantitated using RT–PCR based on a previously described method (Taylor *et al*, 1998). Total RNA was isolated from frozen liver as described above. The primer pairs for UCP-2 were 5′-TAGCAGGAAATCAGAATCAT-3′ and 5′-AAGTGGCAAGGGAGGTCATC-3′ (Genebank: U69135), which generated a 668-bp product. The primers for creating UCP-2 competitor RNA were 5′- AATTTAATACGACTCACTATAGGGATAGCAGGAAATCAGAATCAT-3′ and 5′-CTCGTGCAATGGTCTTGTAG-3′ in an RT–PCR to produce a DNA fragment containing a 128-bp deletion and having an additional 25 bases that correspond to a T7 RNA polymerase-recognition sequence at its 5′ end. Competitor RNA was produced using a T7 RNA polymerase kit (Boehringer-Mannheim) and quantitated using optical density at 260 nm. Six serial two-fold dilutions were prepared containing known concentrations of the competitor for subsequent RT–PCR.

For cDNA template synthesis, 0.5 μg of total RNA and one of the dilutions of competitor RNA in a total volume of 27 μl 0.1% (w v^−1^) diethylpyrocarbonate (DEPC) water was incubated at 65°C for 10 min, then chilled on ice for 2 min. The RNA solution, together with 0.5 μg oligo dT primer and DEPC water, was added to the reaction tube with First-Strand Beads (Pharmacia Biotech, St. Albans, UK) which contained 50 mM Tris (pH 8.3), 75 mM KCl, 7.5 mM DTT, 10 mM MgCl_2_, 0.08 mg ml^−1^ BSA and 2.4 mM each of dATP, dCTP, dGTP and dTTP, and murine reverse transcriptase in a final volume of 33 μl. The contents of the tube were mixed and incubated at 37°C for 1 h.

PCR was performed on a thermal cycler (Hybaid, Ashford, UK), using 2 μl of template cDNA and PCR reaction mixture (Pharmacia Biotech) which gives final concentrations of 1.5 units of Taq polymerase, 10 mM Tris-HCl (pH 9.0), 50 mM KCl, 1.5 mM MgCl_2_, 200 μM of each dNTP and 20 pM of each primer in a final volume of 25 μl. Co-amplification of UCP-2 and the competitor was initiated by one cycle of DNA denaturation for 4 min at 94°C, followed by 35 cycles of denaturing for 1 min at 94°C, annealing at 57.5°C for 1 min and extension for 1 min at 72°C. Finally, an extension for 10 min at 72°C was performed.

Control reactions containing all components except RNA or RNA only were carried out to show that the RNA (both competitor and target) had no DNA contamination. The target and competitor PCR products were sequenced in both orientations and contained the expected sequences and experimentally constructed deletions.

For analysis of results, 10 μl of PCR products were separated in a 1.5% (w v^−1^) agarose gel containing ethidium bromide. The gel images were recorded using a computerized digital camera under UV transillumination and the intensity of bands analyzed using 1D image analysis software (Kodak Digital Science, Eastman Kodak Company, Rochester, NY, USA). The ratio of intensity (competitor/target) was plotted against the six known dilutions of competitor for each sample. The amount of sample RNA corresponds to the amount of competitor when the ratio of competitor to target is 1.0.

### Western blotting

Increases in mRNA of UCPs have been reported under some conditions in the absence of rises in the respective UCP protein levels (Sivitz *et al*, 1999). We therefore used Western blotting to measure UCP-1 and -2 concentrations. UCP-3 could not be measured because a suitable antibody is not available. Mitochondria were isolated from BAT and liver of mouse. Samples containing 30 μg mitochondrial protein were mixed with equal volumes of 2× SDS loading buffer, incubated at 90°C for 5 min, and separated by electrophoresis on 12% SDS-polyacrylamide gels. Proteins were then transferred to nitrocellulose membranes (Hybond C, Amersham Inc, Bucks, UK) and immunological detection was performed using a rabbit affinity-pure UCP-1 antiserum (Autogenbioclear, Wilts, UK) at a 1 : 1000-fold dilution, or a rabbit affinity-pure UCP-2 antiserum (Calbiochem, San Diego, CA, USA) at a 1 : 1000-fold dilution. Blots were then incubated with a goat anti-rabbit secondary antibody conjugated to horseradish peroxidase (DAKO A/S, Glostrop, Denmark). Detection was by using enhanced chemiluminescence (ECL; Amersham Inc, Bucks, UK). The sizes of the proteins detected were estimated using protein rainbow molecular-mass standards (Amersham Inc, Bucks, UK). Autoradiographs and ECL signals were quantitated by scanning densitometry.

### Statistical analyses

Data are expressed as mean±s.e.m. Differences between LMF- treated and control groups were analyzed by the unpaired Student's *t*-test using ARCUS statistical software (Medical Computing, Aughton, UK). Differences were considered as significant if *P*<0.05.

## RESULTS

### LMF reduces body weight and fat mass

Body weight, composition and metabolic data are shown in Table 1

**Table 1:**
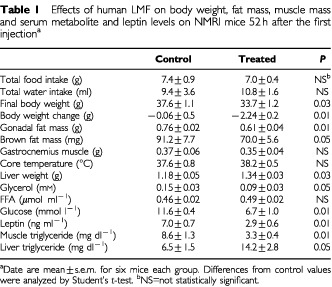
Effects of human LMF on body weight, fat mass, muscle mass and serum metabolite and leptin levels on NMRI mice 52 h after the first injection^a^

**Figure 2:**
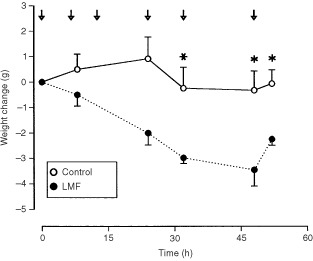
Body weight changes in LMF-treated and vehicle-treated control mice. Arrows indicate time-point of LMF injection. *P<0.01; LMF *vs* controls. Data are mean±s.e.m. for six mice per group.

. As illustrated in Figure 2

, LMF injection caused rapid weight loss, reaching 10% (*P*=0.03) below controls at 52 h. Specifically, LMF-treated mice showed significant reductions in both gonadal fat mass (20%, *P*<0.01) and interscapular BAT (23%, *P*<0.01), whereas skeletal muscle mass was unchanged. Food and water consumption were not significantly altered. Core temperature 4 h after the last LMF injection was slightly higher (+0.6±0.2°C) in LMF-treated mice, but this rise fell short of statistical significance.

### LMF decreases plasma leptin in proportion to reduction of fat mass

As leptin secreted from adipose tissue regulates body adiposity, we therefore tested the lipolytic effects of LMF on leptin. Plasma leptin levels were significantly decreased by 59% below controls in LMF-treated mice (*P*<0.01; Table 1) and this reduction was in proportion to the loss of body fat : plasma leptin was positively correlated with gonadal fat mass across both control and experimental groups (*r*=0.83, *P*<0.01).

### LMF upregulates UCP-1, -2 and -3 mRNA

**Figure 3:**
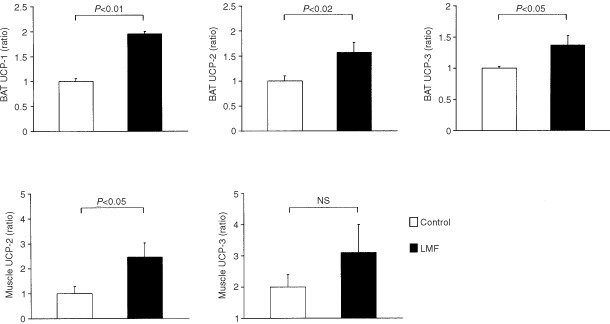
Effect of LMF treatment on mRNA levels of UCP-1, -2 and -3 in BAT, and of UCP-2 and -3 in skeletal muscle. Mice were injected with LMF and tissues were removed at 52 h; control mice received vehicle injections. Total RNA was extracted from mouse BAT or skeletal muscle, and the mRNA levels of the UCPs were measured using Northern blotting with chemiluminescence detection. Data are mean±s.e.m. for six mice per group, expressed as percentage of controls (mean=100%).

**Figure 4:**
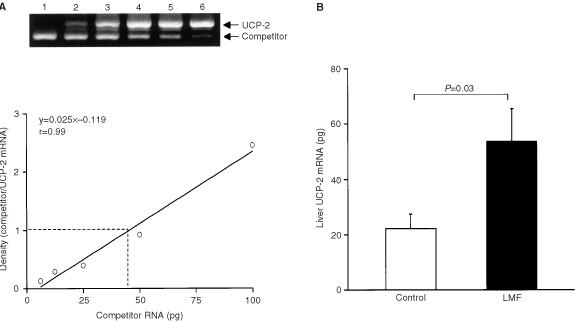
(**A**) Example of RNA quantification by competitive RT–PCR. Lanes 1 to 6, the initial concentrations of the competitor at 100, 50, 25, 12.5, 6.25 and 3.13 pg. The band density ratios between the target and competitor were determined after photographing the agarose gel and plotted *vs* the initial amount of competitor added in the RT–PCR reaction. At the equivalence point (ratio=1), the initial amount of target RNA corresponds to the initial amount of competitor. (**B**) Effect of LMF treatment on UCP-2 mRNA in liver. Mice were injected with LMF and liver was removed at 52 h; control mice received vehicle injections. Data are mean±s.e.m. for six mice per group.

In BAT, LMF treatment significantly increased mRNA levels of UCP-1 (+96%, *P*<0.01), -2 (+57%, *P*=0.02) and -3 (+37%, *P*<0.05). LMF also stimulated UCP-2 expression in muscle (+146%, *P*<0.05); muscle UCP-3 was also increased (+110%), but this rise failed to reach statistical significance (*P*=0.18) ( Figure 3

). Liver UCP-2 mRNA and its competitor mRNA were detectable and quantified by RT–PCR ( Figure 4A

). There was a significant increase in liver UCP-2 mRNA expression in LMF-treated mice compared with vehicle-treated controls (+142%, *P*=0.03; Figure 4B).

### LMF increases protein levels of UCP-1 in BAT and UCP-2 in liver

**Figure 5:**
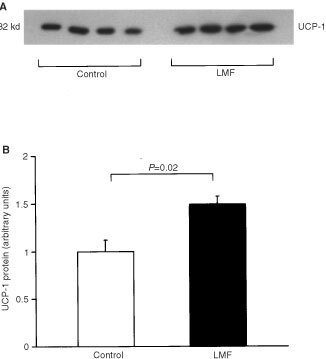
(**A**) Western blot showing UCP-1 concentrations in mitochondrial preparations of brown adipose tissue from vehicle-treated and LMF-treated mice. (**B**) UCP-1 protein content in control and LMF-treated mice (mean±s.e.m. of the signals shown in **A**).

**Figure 6:**
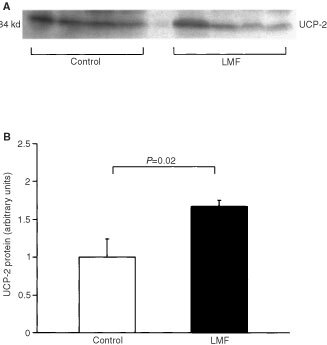
(**A**) Western blot showing UCP-2 content in mitochondrial preparations of liver from vehicle-treated and LMF-treated mice (format as in Figure 5).

Using Western blotting, UCP-1 protein levels in BAT were significantly increased in LMF-treated mice compared with controls (+49%, *P*=0.02; Figure 5

), while UCP-2 protein in liver mitochondrial preparations were also elevated (+67%, *P*=0.02; Figure 6

).

### LMF causes lipid deposition in the liver

**Figure 7:**
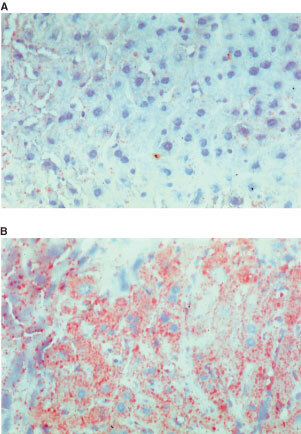
Histology of mouse liver. Fresh frozen tissue from five mice per group was sectioned on a cryostat and stained with Oil Red O. There was marked intracytoplasmic lipid accumulation in the liver from LMF-treated mice (**B**) but little in the vehicle-treated mice (**A**). Hepatocytes from all zones of the hepatic lobule were affected equally.

Haematoxylin and eosin staining of liver sections from LMF-treated mice showed no obvious signs of inflammation or other abnormality. However, Oil Red O staining showed abundant introcytoplasmic microdroplets of lipid deposited in hepatocytes in all zones of the hepatic lobule ( Figure 7A

). By contrast, hepatocytes of control mice contained only sparse and small droplets of lipid (Figure 7B). Caspase 3 immunostaining was no denser or more extensive in the liver of LMF-treated mice than in controls, indicating that apoptosis was not enhanced.

## DISCUSSION

This study confirms the potent lipolytic effects of LMF in normal mice, with marked depletion of body fat but no loss of muscle mass. As well as causing massive adipose tissue catabolism, LMF must somehow act to prevent the resynthesis of triglycerides in this tissue. There is evidence of increased lipid utilization following LMF treatment, including elevation in plasma 3-hydroxybutyrate concentrations and in oxygen uptake by BAT (Hirai *et al*, 1998), but the possibility that other pathways may facilitate LMF-induced fat catabolism in other tissues has not been explored. Here, we investigated the possible involvement of UCP-1, -2 and -3.

We found that LMF administration stimulates UCP-1 mRNA expression in BAT, suggesting that it is at least partly responsible for the UCP-1 upregulation observed in MAC16-bearing mice (Bing *et al*, 2000). The elevation of UCP-1 gene expression also leads to an increase in the relative abundance of UCP-1 protein in the BAT mitochondria. These observations are consistent with the finding that LMF treatment enhances oxygen consumption by BAT, suggesting an increase in BAT thermogenesis. The stimulatory effects of LMF on UCP-1 expression (and on UCP-2 and -3 in other tissues) could be direct and/or indirect actions of LMF. The uncoupling mechanism in BAT mitochondria is controlled by sympathetic afferents via β_3_-adrenoceptors located on the brown adipocytes (Ricquier and Bouillaud, 2000); intriguingly, a recent *in vitro* study has suggested that the lipolytic effect of LMF is mediated through the β_3_-adrenoceptor (Russell *et al*, 2000). Thus, it is possible that LMF upregulates expression of BAT UCP-1 directly via β_3_ receptors. On the other hand, it has been proposed that uncoupling of BAT mitochondria is activated by FFA which stimulate UCP-1 as well as serving as a substrate for oxidation (Trayhurn, 1993; Boss *et al*, 2000). Addition of FFA to isolated brown adipocytes mimics the stimulatory effects of catecholamines on respiration (Prusiner *et al*, 1968), while stimulatory effects of both norepinephrine and FFA are absent in brown adipocytes from UCP-1-ablated mice (Nedergaard *et al*, 1999). As yet, direct actions of LMF on UCP expression in these tissues *in vitro* has not been investigated.

We also found that LMF administration induced UCP-2 gene expression in skeletal muscle, brown fat and liver, and UCP-3 expression in BAT. UCP-2 and -3 are implicated in the regulation of lipids as a fuel substrate in adipose tissue and skeletal muscle in rodents as well as in humans (Samec *et al*, 1998a; Khalfallah *et al*, 2000; Nisoli *et al*, 2000); accordingly, our findings support a role for UCP-2 and -3 in enhancing the removal by those tissues of lipids mobilized by LMF. LMF administration also caused massive lipid deposition in the liver that was associated both with induction of UCP-2 mRNA and mild increased UCP-2 protein abundance in that organ. UCP-2 has recently been implicated in hepatic lipid utilization, as exposure to intralipid emulsions leads to increases in UCP-2 mRNA and UCP-2 protein levels in cultured hepatocytes (Cortez-Pinto *et al*, 1999). Lipid oxidation is known to generate intracellular reactive oxygen species (ROS) that can cause cell death, while the uncoupling of respiration can be a powerful mechanism that limits ROS formation. Inhibiting BAT UCP-1 or disruption of UCP-2 gene in mice stimulates ROS production, while both the ROS-inducing agent *tert*-butyl hydroperoxide (TBHP) and tumour necrosis factor-α (TNF-α) can induce hepatocyte UCP-2 mRNA; this suggests that UCPs may also serve as an antioxidant defence mechanism (Negre-Salvayre *et al*, 1997; Cortez-Pinto *et al*, 1999; Lee *et al*, 1999; Arsenijevic *et al*, 2000). Perhaps consistent with this is the observation that fatty livers from *ob/ob* mice or ethanol-fed lean mice show up-regulation of hepatic transcripts for UCP-2, while hepatocyte death is not increased (Rashid *et al*, 1999). In the present study, induction of UCP-2 in liver when there is an excessive lipid accumulation caused by LMF could also limit ROS production, thereby preventing cell death. Indeed, we found no evidence of excess apoptosis in the fatty liver of mice treated with LMF.

Taken together, our findings suggest that uncoupling proteins play a permissive role in enhanced lipolysis in cancer cachexia. Induction of UCP-1, -2 and -3 expression by tumour-derived LMF probably provides a mechanism for excessive lipid disposal, which in turn facilitates the fat catabolic cascade in malignancy. UCP induction may help to protect these tissues against the oxidative damage that would result from enhanced FFA oxidation.
